# Construction of 1D/2D *α*-Fe_2_O_3_/SnO_2_ Hybrid Nanoarrays for Sub-ppm Acetone Detection

**DOI:** 10.34133/2020/2196063

**Published:** 2020-02-13

**Authors:** Huimin Gong, Changhui Zhao, Gaoqiang Niu, Wei Zhang, Fei Wang

**Affiliations:** ^1^School of Microelectronics, Southern University of Science and Technology, Shenzhen 518055, China; ^2^Department of Electrical and Electronic Engineering, Southern University of Science and Technology, Shenzhen 518055, China

## Abstract

Exhaled acetone is one of the representative biomarkers for the noninvasive diagnosis of type-1 diabetes. In this work, we have applied a facile two-step chemical bath deposition method for acetone sensors based on *α*-Fe_2_O_3_/SnO_2_ hybrid nanoarrays (HNAs), where one-dimensional (1D) FeOOH nanorods are in situ grown on the prefabricated 2D SnO_2_ nanosheets for on-chip construction of 1D/2D HNAs. After annealing in air, ultrafine *α*-Fe_2_O_3_ nanorods are homogenously distributed on the surface of SnO_2_ nanosheet arrays (NSAs). Gas sensing results show that the *α*-Fe_2_O_3_/SnO_2_ HNAs exhibit a greatly enhanced response to acetone (3.25 at 0.4 ppm) at a sub-ppm level compared with those based on pure SnO_2_ NSAs (1.16 at 0.4 ppm) and pure *α*-Fe_2_O_3_ nanorods (1.03 at 0.4 ppm), at an operating temperature of 340°C. The enhanced acetone sensing performance may be attributed to the formation of *α*-Fe_2_O_3_–SnO_2_ n-n heterostructure with 1D/2D hybrid architectures. Moreover, the *α*-Fe_2_O_3_/SnO_2_ HNAs also possess good reproducibility and selectivity toward acetone vapor, suggesting its potential application in breath acetone analysis.

## 1. Introduction

As a potential alternative for the noninvasive diagnosis of disease, exhaled breath analysis has been proposed and developed over the past decades [1–3]. The exhaled breath of human beings includes not only nitrogen, oxygen, carbon dioxide, nitric oxide, and water vapor but also a mixture of volatile organic compounds (VOCs) and some other nonvolatile molecules. Encouragingly, a few of them have been regarded as biomarkers to diagnose diseases ([Supplementary-material supplementary-material-1]). For example, formaldehyde (lung cancer) [4], toluene (lung cancer) [5], ammonia (hemodialysis) [6], H_2_S (halitosis) [7], isoprene (heart disease) [8], benzene (smoker) [9], and pentane (acute asthma) [10] at few dozens to few thousands of ppb are known as biomarkers for patients. Researchers have also found that exhaled acetone can intuitively correlate with type-1 diabetes, which may exceed 1.8 ppm (only 0.3–0.9 ppm for healthy people) [1, 11]. Therefore, an ultrasensitive acetone sensor is of great importance to detect acetone vapor at a sub-ppm level.

Metal oxide semiconductors (MOXs), such as SnO_2_, ZnO, *α*-Fe_2_O_3_, CuO, and NiO, have been widely explored in the field of gas detection owing to their simple and cost-effective synthesis, high sensitivity, and good stability. Among these MOXs, *α*-Fe_2_O_3_ is a multifunctional n-type semiconductor with a direct bandgap (*E*_g_ = 2.2 eV at 300 K) that has been intensively investigated in the field of gas sensing [12–15]. Several effective strategies have been designed to improve the gas sensing properties of these MOXs, such as doping, surface modification, porous/hollow structures, and hierarchical architectures [16–18]. Recently, construction of hybrid nanostructures is rapidly emerging as a fascinating strategy that combines different MOXs with precise control of their morphologies, such as hollow ZnO/ZnFe_2_O_4_ heterostructures that were synthesized by growing ultrathin ZnFe_2_O_4_ nanosheets on the outer surface of ZnO hollow microspheres [19], NiO nanoparticle-decorated SnO_2_ nanosheets [20], CuO nanosheets/ZnO nanorods (NRs) [21], and Fe_2_O_3_ nanoparticle-decorated CuO NRs [22]. For this purpose, the rational combination of SnO_2_ and *α*-Fe_2_O_3_ has been proven to improve their gas sensing performances ([Supplementary-material supplementary-material-1]). The results show that the *α*-Fe_2_O_3_/SnO_2_ composites present excellent sensing performances to acetone [23, 24], ethanol [25–30], toluene [31], and LPG [32, 33]. Moreover, their gas sensing properties can be largely affected by the size and shape of nanobuilding blocks (*α*-Fe_2_O_3_ and SnO_2_). As far as we know, only a few reports about *α*-Fe_2_O_3_–SnO_2_ system have concerned on the detection of ultralow concentrations of acetone.

In this work, we report a two-step chemical bath deposition (CBD) method to construct the *α*-Fe_2_O_3_/SnO_2_ hybrid nanoarrays (HNAs) on-chips with subsequent annealing in air. The one-dimensional (1D) *α*-Fe_2_O_3_ NRs are distributed homogenously on the surface of the 2D SnO_2_ nanosheets to construct novel 1D/2D HNAs. In comparison with pure SnO_2_ NSAs and *α*-Fe_2_O_3_ NRs, the *α*-Fe_2_O_3_/SnO_2_ HNAs show a dramatically enhanced response to acetone (down to sub-ppm). Moreover, the *α*-Fe_2_O_3_/SnO_2_ HNAs also possess a superior selectivity to acetone against other interfering gases (formaldehyde, toluene, benzene, and ammonia). A possible sensing mechanism based on the formation of *α*-Fe_2_O_3_–SnO_2_ n-n heterostructure is proposed.

## 2. Results and Discussion

### 2.1. Morphological Characteristics

**Figure 1:**
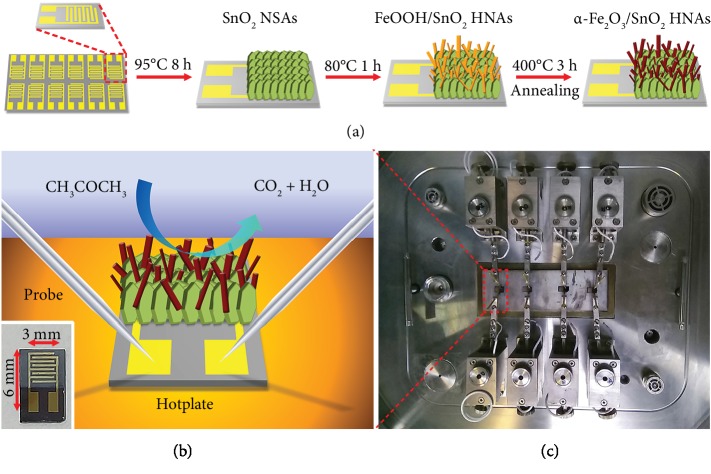
Schematic diagrams of (a) synthesis process of *α*-Fe_2_O_3_/SnO_2_ HNAs and (b) gas sensing measurement platform; the inset shows the digital photograph of the *α*-Fe_2_O_3_/SnO_2_ HNAs gas sensing chips. (c) A photograph of the test platform of CGS-4TPs.

As illustrated in Figure 1(a), a chip with interdigital Au electrodes (200 *μ*m lines separated by 200 *μ*m gaps) was fabricated on a (100) silicon substrate with a 2 *μ*m thermally grown SiO_2_ layer, and the SnO_2_ NSAs were prepared with an on-chip growth method similar to our previous work [34]. The chip was vertically dipped into the mixed solution (containing Sn^2+^ and CO(NH_2_)_2_) during this process, and then the prefabricated SnO_2_ NSAs were immersed in another aqueous solution (containing Fe^2+^ and CO(NH_2_)_2_) for depositing FeOOH NRs on SnO_2_ NSAs. After annealing in air, the as-prepared sensors were placed on a CGS-4TP gas sensing measurement system. A schematic of the gas sensing measurement systems used in this work is illustrated in [Supplementary-material supplementary-material-1]. Figure 1(b) presents a schematic diagram of the test platform used in this work. A hotplate was used to adjust the operating temperature, and two pins of a sensor were connected with a pair of probes. A digital photograph of the gas sensing chip (3 mm × 6 mm in size) with *α*-Fe_2_O_3_/SnO_2_ HNAs is given in the inset of Figure 1(b). During the test, four sensors were measured simultaneously, as shown Figure 1(c), and the electrical resistance of each sensor was recorded.

**Figure 2:**
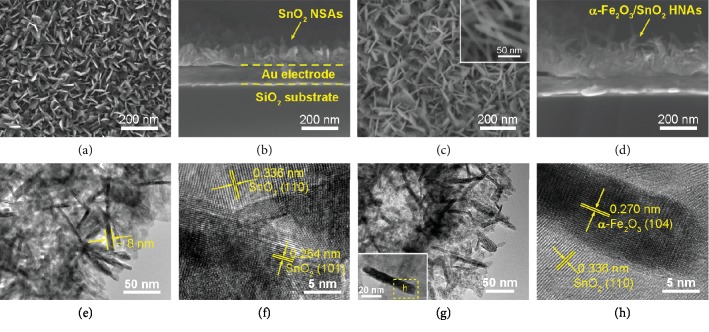
Top view SEM images of (a) SnO_2_ NSAs and (c) *α*-Fe_2_O_3_/SnO_2_ HNAs. Cross-sectional SEM images of (b) SnO_2_ NSAs and (d) *α*-Fe_2_O_3_/SnO_2_ HNAs. The inset of (c) shows a SEM close-up image of *α*-Fe_2_O_3_ NRs. (e) TEM and (f) HRTEM images of the pure SnO_2_ NSAs. (g) TEM and (h) HRTEM images of *α*-Fe_2_O_3_/SnO_2_ HNAs. The inset of (g) shows an individual *α*-Fe_2_O_3_ rod grown on the surface of SnO_2_ nanosheet.

Figure 2 shows the morphologies of as-prepared pure SnO_2_ NSAs and *α*-Fe_2_O_3_/SnO_2_ HNAs. It can be seen from Figure 2(a) that the pure SnO_2_ NSAs are composed by oriented growth of nanosheets, where the adjacent SnO_2_ nanosheets will interconnect with each other and form a semiopen network. From the cross-sectional scanning electron microscope (SEM) images of SnO_2_ NSAs (Figure 2(b)), the flake-like SnO_2_ stands vertically on the chip with a uniform film thickness (~100 nm), and the SnO_2_ NSAs are robustly adhered to the substrate. After the growth of *α*-Fe_2_O_3_ NRs, it is obvious that the surface morphology of *α*-Fe_2_O_3_/SnO_2_ HNAs is much different from that of the pure SnO_2_ NSAs. As shown in Figure 2(c), numerous ultrathin *α*-Fe_2_O_3_ NRs are homogenously distributed among the interconnected SnO_2_ NSAs. A SEM close-up image of *α*-Fe_2_O_3_ NRs (inset Figure 2(c)) reveals that the diameter of NRs ranges from 9 nm to 20 nm, and the average diameter is about 12.7 nm. Otherwise, for the second-step (CBD method), the *α*-Fe_2_O_3_ NRs tend to form irregular aggregates without a substrate ([Supplementary-material supplementary-material-1]). The cross-sectional SEM images of *α*-Fe_2_O_3_/SnO_2_ HNAs in Figure 2(d) further indicate that the 1D *α*-Fe_2_O_3_ NRs are in situ grown on the surface of 2D SnO_2_ nanosheets, and novel 1D/2D hybrid nanoarrays can be achieved by a facile two-step CBD method. At the same time, the average film thickness of *α*-Fe_2_O_3_/SnO_2_ HNAs increases up to 220 nm.

To get further insight into the definite morphology of pure SnO_2_ NSAs and *α*-Fe_2_O_3_/SnO_2_ HNAs, transmission electron microscope (TEM) images were taken from the scraped-off products. As shown in Figure 2(e), SnO_2_ NSAs made up of interconnecting flakes with a thickness of <10 nm are obtained. Because of the vertical direction of growth, the thickness of a SnO_2_ nanosheet can be easily measured in Figure 2(e) (marked by arrows, ~8 nm). These 2D nanosheets have an edge length of tens of nanometers, which agree well with the SEM observation (Figure 2(a)). A high-resolution TEM (HRTEM) image (Figure 2(f)) reveals the fringe patterns in SnO_2_ NSAs, and the *d*-spacings of 0.264 and 0.336 nm are assigned to the interplanar distances of (101) and (110) planes of rutile SnO_2_, respectively.

For *α*-Fe_2_O_3_/SnO_2_ HNAs, the overall TEM image (Figure 2(g)) indicates that the hybrid composites are constructed by interconnected 2D nanosheets and some disordered 1D nanorods with respect to their different structural features. The inset of Figure 2(g) shows an individual nanorod grown on the surface of the nanosheet. The diameter of the rod is around 9 nm, and the length is estimated to be 57 nm. Figure 2(h) provides the HRTEM image of the selected region from the inset of Figure 2(g) (marked by a dashed rectangle). The lattice fringes with *d*-spacings of 0.270 and 0.336 nm can be indexed to the (104) plane of *α*-Fe_2_O_3_ and (110) plane of SnO_2_, respectively. These results further confirm the construction of the 1D/2D hybrid nanostructure of *α*-Fe_2_O_3_/SnO_2_ HNAs.

**Figure 3:**
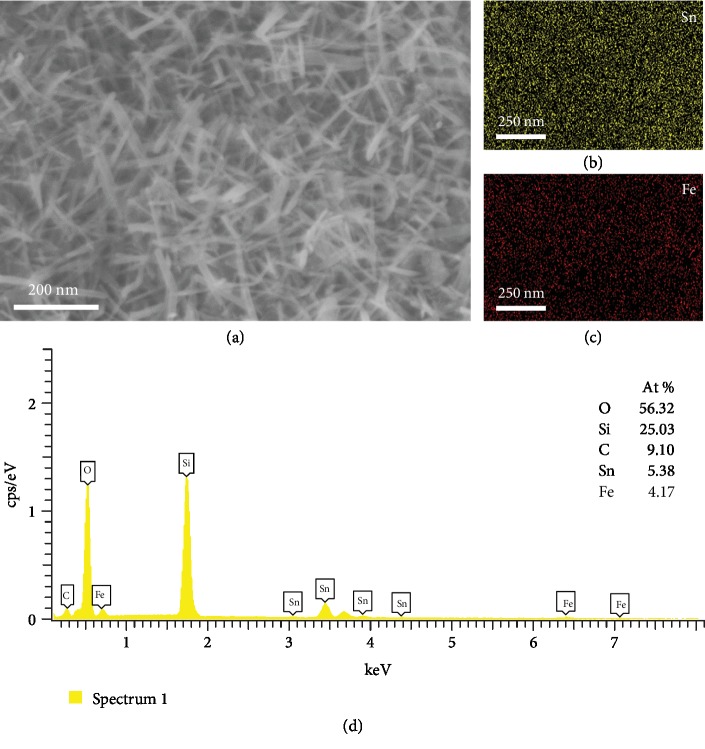
(a) SEM image, (b–c) the corresponding EDS mapping images, and (d) EDS spectrum of *α*-Fe_2_O_3_/SnO_2_ HNAs.

The chemical composition of the samples was identified by EDS and XPS analysis. The EDS mapping and spectrum of *α*-Fe_2_O_3_/SnO_2_ HNAs are depicted in Figures 3(a)–3(c) and 3(d), respectively. Sn (Figure 3(b)) and Fe (Figure 3(c)) elements were distributed randomly and uniformly on the substrate, which was in agreement with the fact that the hybrid composites were constructed by hybrid *α*-Fe_2_O_3_ and SnO_2_. The existence of Sn and Fe elements was confirmed by the EDS characterization, and the atomic percentages of Sn and Fe were measured to be 5.38% and 4.17%, respectively.

**Figure 4:**
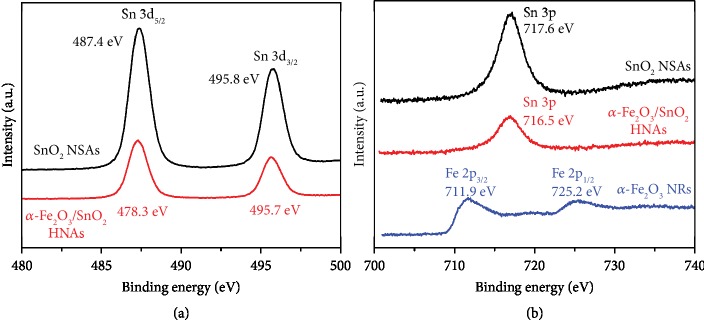
The high-resolution XPS spectra of SnO_2_ NSAs, *α*-Fe_2_O_3_ NRs, and *α*-Fe_2_O_3_/SnO_2_ HNAs: (a) Sn 3d and (b) Fe 2p.

Moreover, XPS analysis was used to obtain more information about the chemical valences of our samples.  Figure 4(a) displays the high-resolution Sn 3d spectra of SnO_2_ NSAs and *α*-Fe_2_O_3_/SnO_2_ HNAs. In the pure SnO_2_ NSAs, the two peaks centered at 487.4 and 495.8 eV can be ascribed to the peaks of Sn 3d_5/2_ and Sn 3d_3/2_, respectively, which are in good agreement with Sn^4+^. With the modification of *α*-Fe_2_O_3_, a slight negative shift of the binding energies is observed in *α*-Fe_2_O_3_/SnO_2_ HNAs, shifting to 487.3 and 495.7 eV, respectively, as a result of the formation of *α*-Fe_2_O_3_/SnO_2_ heterojunction interface. In the spectrum of Fe 2p in Figure 4(b), interference peaks are detected at 717.6 eV in SnO_2_ NSAs and 716.5 eV in *α*-Fe_2_O_3_/SnO_2_ HNAs, which come from the Sn 3p peak. The Fe 2p peaks are not found in *α*-Fe_2_O_3_/SnO_2_ HNAs due to the strong interference peak. In comparison, the two peaks at 711.9 and 725.2 eV detected in pure *α*-Fe_2_O_3_ NRs are attributed to Fe 2p_3/2_ and Fe 2p_1/2_, respectively, corresponding to Fe^3+^ in *α*-Fe_2_O_3_.

### 2.2. Gas Sensing Properties

**Figure 5:**
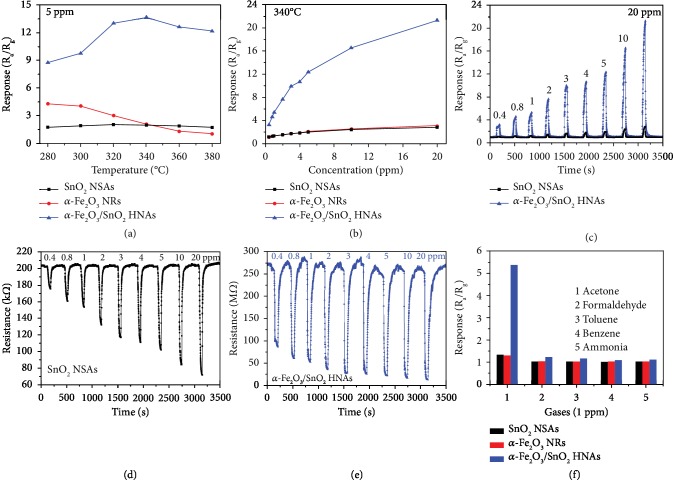
(a) Sensor responses of the pure SnO_2_ NSAs, *α*-Fe_2_O_3_ NRs, and *α*-Fe_2_O_3_/SnO_2_ HNAs toward 5 ppm acetone as a function of operating temperature (280-380°C). (b) Sensor responses *vs.* acetone concentration (0.4-20 ppm) at 340°C. The corresponding transient response curves of (c) SnO_2_ NSAs and *α*-Fe_2_O_3_/SnO_2_ HNAs. Resistance curves of (d) SnO_2_ NSAs and (e) *α*-Fe_2_O_3_/SnO_2_ HNAs at 340°C. (f) Selectivity of the sensors to various gases (1 ppm) at 340°C.

As is well-known, the gas sensing properties of MOXs are highly dependent on the operating temperature. To confirm it, the as-prepared gas sensors were examined at various temperatures (280-380°C) toward 5 ppm acetone. The sensor response is defined as *R*_a_/*R*_g_, where *R*_a_ and *R*_g_ are the sensor resistance in air and target gas, respectively. As shown in Figure 5(a), the response of *α*-Fe_2_O_3_/SnO_2_ HNA-based sensor increases with the increase in operating temperature and reaches its maximum value (13.63) at 340°C, then decreases with the further increase of operating temperature. Therefore, 340°C can be chosen as the optimum operating temperature of *α*-Fe_2_O_3_/SnO_2_ HNAs. Differently, the pure SnO_2_ NSA-based sensor exhibits no obvious variation over the whole temperature range (2.00, at 340°C). The pure *α*-Fe_2_O_3_ NR-based sensor shows a monotonic decrease of the response with an increase in operating temperature, and the highest response value is about 4.28 at 280°C. It is clear that the *α*-Fe_2_O_3_/SnO_2_ HNA-based sensor displays the highest response in the three sensors, revealing that the acetone sensing properties of SnO_2_ NSAs can be significantly enhanced by the modification of *α*-Fe_2_O_3_ NRs.

Figure 5(b) gives the acetone sensing properties of the above three sensors at the same operating temperature of 340°C. It is clear that the sensor response increases with the acetone concentration ranging from 0.4 to 20 ppm for each sensor. Especially, in the case of *α*-Fe_2_O_3_/SnO_2_ HNAs, the response increases rapidly over the whole concentration range, which is rather different from the other two sensors. The response values of *α*-Fe_2_O_3_/SnO_2_ HNAs are 3.25, 4.64, 5.37, 7.68, 9.91, 10.69, 12.34, 16.55, and 21.26 toward 0.4, 0.8, 1, 2, 3, 4, 5, 10, and 20 ppm acetone, respectively. In comparison, the response values of pure SnO_2_ NSAs and *α*-Fe_2_O_3_ NRs toward acetone can be as low as 1.16 and 1.03, respectively, at a concentration of 0.4 ppm, and their values are still less than 3.1 even toward 20 ppm acetone. Therefore, the *α*-Fe_2_O_3_/SnO_2_ HNA-based sensor exhibits the highest response values toward acetone in the three sensors, indicating the improvement of sensitivity.

Figure 5(c) plots the corresponding transient response curves of the pure SnO_2_ NSA- and *α*-Fe_2_O_3_/SnO_2_ HNA-based sensors over an acetone concentration range of 0.4 to 20 ppm recorded at 340°C. Obviously, these response curves present a sharp increase upon acetone exposure and can recover to their original values in air. In accordance with these, the sensor resistance curves are shown in Figures 5(d) and 5(e). Amongst them, the *α*-Fe_2_O_3_/SnO_2_ HNA-based sensor exhibits a higher resistance in air (*R*_a_, 282.683 MΩ, Figure 5(e)) than that of pure SnO_2_ NSAs (204.05 kΩ, Figure 5(d)) and *α*-Fe_2_O_3_ NRs (132.298 MΩ, [Supplementary-material supplementary-material-1]). Upon exposure to acetone gas, the sensor resistance quickly decreases as expected and then recovers to its *R*_a_ after being exposed to air. *R*_g_ decreases monotonically with the increase of acetone concentration; in other words, the sensor response increases (refer to Figure 5(c) and [Supplementary-material supplementary-material-1]).

The response and recovery times (*t*_res_ and *t*_rec_) are very important parameters for high-performance gas sensors. The response time *t*_res_ (or recovery time *t*_rec_) is defined as the time required to reach 90% resistance change when the sensor is exposed to target gas (or air). As shown in [Supplementary-material supplementary-material-1], the *α*-Fe_2_O_3_/SnO_2_ HNA-based sensor shows a faster *t*_res_ (14 s, at 1 ppm) at 340°C compared with that of the SnO_2_ NSAs (37 s, at 1 ppm). On the contrary, the *t*_rec_ of the *α*-Fe_2_O_3_/SnO_2_ HNA-based sensor always exceeds one minute (62–159 s, in the range 0.4–20 ppm), which is apparently higher than that of the SnO_2_ NSA-based sensor (22–34 s, in the range 0.4–20 ppm). According to the previous studies, the vertically ultrathin SnO_2_ NSAs can provide as much surface area as possible to adsorb gas molecules and facilitate the adsorption/desorption of the acetone gas. When the *α*-Fe_2_O_3_ NRs were introduced, the branched *α*-Fe_2_O_3_ NRs on the surface of SnO_2_ NSAs will adsorb more acetone molecules, making the *α*-Fe_2_O_3_/SnO_2_ HNAs more sensitive and present faster response toward acetone.

To study the selectivity in the above sensors, some interfering gases (formaldehyde, toluene, benzene, and ammonia) were measured at 340°C with a low concentration of 1 ppm. It can be seen in Figure 5(f) that the *α*-Fe_2_O_3_/SnO_2_ HNA-based sensor exhibits higher responses toward all gases than those of pure SnO_2_ NSAs and *α*-Fe_2_O_3_ NRs. Especially, all the sensors obtain their highest responses toward acetone compared with other interfering gases. In the case of the *α*-Fe_2_O_3_/SnO_2_ HNA-based sensor, it shows the highest response toward acetone (5.37), then toward formaldehyde (1.23) and toluene (1.16) and is almost insensitive toward ammonia (1.11) and benzene (1.09). On the other hand, the corresponding responses of pure SnO_2_ NSAs toward above gases are 1.33, 1.02, 1.02, 1.02, and 1.01 in turn (for *α*-Fe_2_O_3_ NRs: 1.30, 1.03, 1.02, 1.03, and 1.03). These results suggest that the *α*-Fe_2_O_3_ NRs, indeed, have a significant impact on the selectivity of the SnO_2_ NSAs toward acetone.

**Figure 6:**
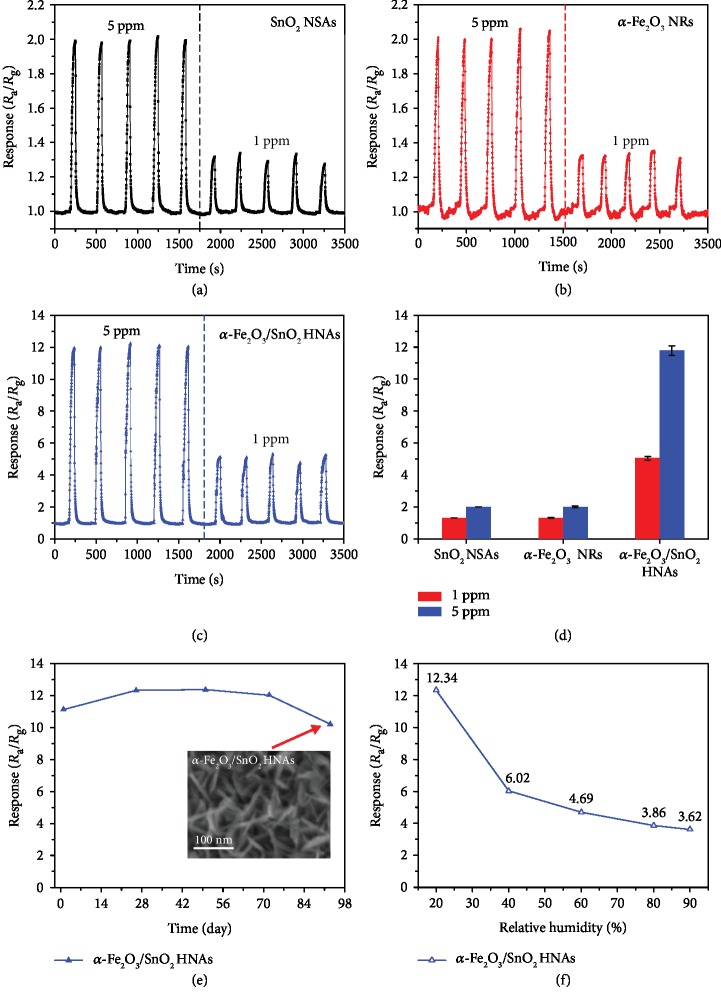
Reproducibility of (a) SnO_2_ NSAs, (b) *α*-Fe_2_O_3_ NRs, and (c) *α*-Fe_2_O_3_/SnO_2_ HNA-based sensors toward acetone (5 ppm and 1 ppm, each of 5 cycles) at 340°C; (d) is the corresponding comparison of the sensor responses. (e) Stability of *α*-Fe_2_O_3_/SnO_2_ HNA-based sensor toward 5 ppm acetone at 340°C for 93 days; the inset is the SEM image of *α*-Fe_2_O_3_/SnO_2_ HNAs taken after 93 days of gas sensing test. (f) Response of *α*-Fe_2_O_3_/SnO_2_ HNA-based sensor to 5 ppm acetone at 340°C under different relative humidity (20–90% RH).

The reproducibility of the sensors at 340°C has been investigated by continuously testing the sensors to 5 ppm and 1 ppm acetone with 5 cycles for each. As shown in Figures 6(a)–6(c), all the sensors maintain their response values without obvious variation (less than 4%) during the cyclic testing, indicating excellent reproducibility of our devices. By comparison, Figure 6(d) illustrates the statistical analysis of the results of sensor responses (for SnO_2_ NSAs sensor: 1.32 ± 0.02 at 1 ppm and 2.00 ± 0.01 at 5 ppm; *α*-Fe_2_O_3_ NRs sensor: 1.33 ± 0.03 at 1 ppm and 2.02 ± 0.06 at 5 ppm; *α*-Fe_2_O_3_/SnO_2_ HNAs sensor: 5.05 ± 0.11 at 1 ppm and 11.80 ± 0.29 at 5 ppm), further demonstrating their robustness as acetone sensors.

To assess the long-term stability of our sensor, we tested the *α*-Fe_2_O_3_/SnO_2_ HNA-based sensor for 93 days toward 5 ppm acetone at 340°C. The mean response of the *α*-Fe_2_O_3_/SnO_2_ HNA-based sensor is 11.62 with a standard deviation estimated to be 0.93 during the whole period, suggesting its stability for acetone detection over a long period. Furthermore, it can be clearly seen that there is no obvious change between the nanostructures of *α*-Fe_2_O_3_/SnO_2_ HNAs before (Figure 2(c)) and after (inset of Figure 6(e)) a number of gas sensing tests. This observation is consistent with the good long-term stability of the *α*-Fe_2_O_3_/SnO_2_ HNA-based sensor.

It is well-known that human exhaled breath is highly humid (RH ≥ 80%) and the existence of water vapor has a significant influence on the gas sensing performance for MOX-based gas sensors. As shown in Figure 6(f), the response of *α*-Fe_2_O_3_ NRs to 5 ppm acetone was measured as a function of relative humidity (20%–90% RH). The responses of *α*-Fe_2_O_3_/SnO_2_ HNAs under 20%, 40%, 60%, 80%, and 90% RH were 12.34, 6.02, 4.69, 3.86, and 3.62, respectively. Obviously, the response of *α*-Fe_2_O_3_/SnO_2_ HNAs is highly dependent on relative humidity, and some available approaches (such as employing water filtering membranes) are needed to eliminate the influence of water vapor.

Considering the previous reports in Table 1 [11, 35–38], the *α*-Fe_2_O_3_/SnO_2_ HNA-based sensor in this work possesses relatively medium sensitivity (or operating temperature). We can conclude that the acetone sensing properties of MOXs can be further enhanced by constructing heterostructures or modifying with noble metals. As mentioned before, the acetone detection capability (or resolution) for the diagnosis of diabetes mellitus should be as low as sub-ppm, all of which need sufficient and reliable sensors for acetone. In this sense, the high sensitivity, good selectivity, and excellent reproducibility of the *α*-Fe_2_O_3_/SnO_2_ HNA-based sensor imply that it can potentially be used for breath acetone analysis.

### 2.3. Sensing Mechanism

**Figure 7:**
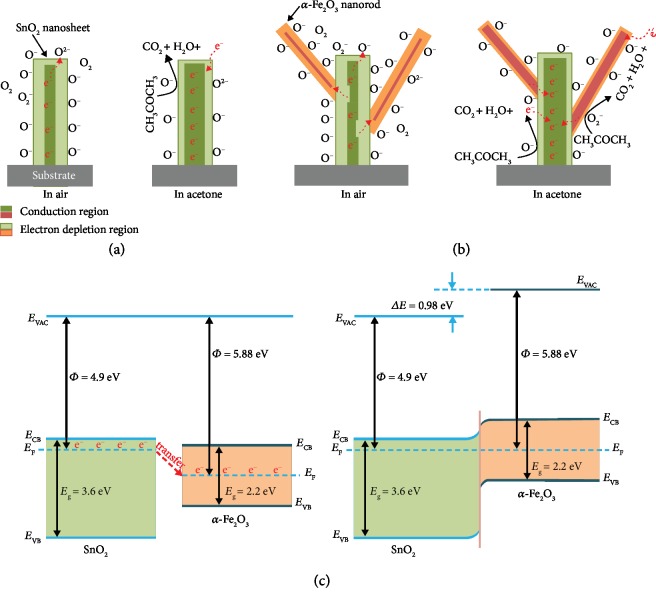
Schematic illustration of the acetone sensing mechanism of (a) the pure SnO_2_ NSAs and (b) *α*-Fe_2_O_3_/SnO_2_ HNAs (not drawn to scale). (c) Energy band diagrams of *α*-Fe_2_O_3_/SnO_2_ system before and after equilibrium. *E*_VAC_: the vacuum level; *E*_F_: Fermi level; *E*_CB_: the bottom of conduction band; *E*_VB_: the top of valence band; *E*_g_: band gap.

For n-type MOXs (SnO_2_ and *α*-Fe_2_O_3_), their acetone sensing mechanisms can be briefly understood as the reaction between the adsorbed oxygen species and acetone molecules on the active sites of sensitive materials. [24] In general, an electron depletion layer (EDL) can be formed on the near surface of SnO_2_ nanosheets (Figure 7(a)) owing to the adsorbed oxygen species (O^–^, O_2_^–^, and O^2–^) after exposing to air and makes the absorbed oxygen species capture free electrons from the conduction band of SnO_2_. This results in a decrease of electron concentration (or width of EDL) and thus a relatively high resistance in air atmosphere. The generation and transformation processes of the oxygen species at different operating temperatures have the following expressions [39]:

(1) $$\begin{array}{c} {\text{O}}_{2}\left(\text{gas}\right)⟷{\text{O}}_{2}\left(\text{ads}\right)\\ {\text{O}}_{2}+{\text{e}}^{-}⟷{{\text{O}}_{2}}^{-}\\ {{\text{O}}_{2}}^{-}+{\text{e}}^{-}⟷2{\text{O}}^{-}\\ {\text{O}}^{-}+{\text{e}}^{-}⟷{\text{O}}^{2-} \end{array}$$

On the contrary, upon exposure to reducing gases such as acetone, acetone molecules will react with the absorbed oxygen species, as expressed by Equation (2) and release free electrons back to the SnO_2_ nanosheets. Hence, the electron concentration increase will cause an increase in conductivity (or a decrease in sensor resistance), and the width of EDL also becomes broader. According to the SEM and TEM observations, the vertically distributed SnO_2_ nanosheets connect with each other to construct an interlaced electron transport network on the substrate. However, the modulation mechanism of this type of transport network is not efficient because of the same energy band structure in the pure SnO_2_ NSAs. In other words, the *R*_a_ of the pure SnO_2_ NSA-based sensor is rather low (Figure 5(d)), which is too difficult to obtain dramatic change, especially at low acetone concentrations.

(2) $$\begin{array}{c} {\text{CH}}_{3}{\text{COCH}}_{3}+8{\text{O}}^{-}⟶3{\text{CO}}_{2}+3{\text{H}}_{2}\text{O}+8{\text{e}}^{-}\\ {\text{CH}}_{3}{\text{COCH}}_{3}+8{\text{O}}^{2-}⟶3{\text{CO}}_{2}+3{\text{H}}_{2}\text{O}+16{\text{e}}^{-} \end{array}$$

In the case of *α*-Fe_2_O_3_/SnO_2_ HNAs, the sensor exhibits enhanced sensitivity toward acetone, this may be attributed to the following reasons: (1) In the formation of *α*-Fe_2_O_3_–SnO_2_ n–n heterostructures, by combining these two MOXs with different work functions (SnO_2_: *𝛷* = 4.9 eV; *α*-Fe_2_O_3_: *𝛷* = 5.88 eV) [40, 41], the free electrons tend to transfer from the higher side to the lower side, until the equilibrium Fermi level is reached (Figure 7(c)) [26, 42]. In this process, the SnO_2_ nanosheet near the heterostructure interface will lose more electrons, which leads to a broader conduction region in air (Figure 7(b)) [43]. Similar to other reports, the *R*_a_ of the *α*-Fe_2_O_3_/SnO_2_ HNAs in this work is much higher than that of the pure SnO_2_ NSAs.

(2) In the novel 1D/2D *α*-Fe_2_O_3_/SnO_2_ hybrid architectures, when the sensor is exposed to acetone, the stretched-out *α*-Fe_2_O_3_ NRs provide an extra surface area and active sites for the gas adsorption. Thus, more oxygen species and acetone molecules can be adsorbed on the surface of *α*-Fe_2_O_3_/SnO_2_ HNAs (Figure 7(b)), which provides more opportunities for Equation (2). The conduction region in SnO_2_ nanosheets will be broadened as well as a decrease in *R*_g_. On the other hand, the free electrons generated on the surface of *α*-Fe_2_O_3_ NRs will flow to SnO_2_ NSAs and allow a dramatic decrease in the width of the electron depletion region at the interface of the *α*-Fe_2_O_3_/SnO_2_ heterostructure. It may further result in an increase of sensor response toward acetone. So the modulation mechanism of *α*-Fe_2_O_3_/SnO_2_ HNAs becomes more efficient than that of pure SnO_2_ NSAs. Additionally, much hard work is still needed to study the influence of ambient humidity, filter units, and clinical tests, making it more suitable for breath acetone analysis.

## 3. Conclusion

The *α*-Fe_2_O_3_/SnO_2_ HNA-based acetone sensor has been fabricated via a facile two-step on-chip growth (or CBD method) process. The results indicate that the as-prepared sensor presents a well-defined 1D/2D hybrid architecture, where the ultrathin *α*-Fe_2_O_3_ NRs (an average diameter ~12.7 nm) are distributed among the interconnected SnO_2_ NSAs. Gas sensing measurements show that the *α*-Fe_2_O_3_/SnO_2_HNA-based sensor exhibits superior acetone sensing properties (high sensitivity, good reproducibility, and selectivity), even at a sub-ppm level, compared with those of the pure SnO_2_ NSA- and *α*-Fe_2_O_3_ NR-based sensors. The improved acetone sensing performance may be due to the formed *α*-Fe_2_O_3_–SnO_2_ heterostructures and their unique hybrid nanostructures. Our work suggests that the *α*-Fe_2_O_3_/SnO_2_ HNAs can be a promising candidate for sub-ppm acetone detection in breath analysis.

## 4. Materials and Methods

### 4.1. Preparation of *α*-Fe_2_O_3_/SnO_2_ HNAs

In brief, 0.6 mmol SnCl_2_·2H_2_O and 0.8 mmol CO(NH_2_)_2_ were dissolved into 20 mL deionized water and stirred for 15 min at room temperature. Then a piece of chip with several Au electrodes was washed with acetone and ethanol and deionized water for several times, which was afterwards vertically dipped into the above solution and maintained at 95°C for 8 h. After washing and drying at 60°C in an oven, the prefabricated SnO_2_ NSAs were immersed in an aqueous solution (containing 0.1 M FeSO_4_·7H_2_O and 1.0 M CO(NH_2_)_2_) and kept at 80°C for 1 h. Similarly, the chip was washed and dried again at 60°C. The final chip was annealed at 400°C for 3 h in air to achieve *α*-Fe_2_O_3_/SnO_2_ HNAs.

In addition, the pure SnO_2_ NSAs were also annealed under the same conditions. For a pure *α*-Fe_2_O_3_ NR-based sensor, 0.1 M FeSO_4_·7H_2_O and 1.0 M CO(NH_2_)_2_ were mixed and maintained at 80°C for 1 h, and the collected precipitate was dip-coated on the Au electrodes and then annealed at 400°C in air for 3 h.

### 4.2. Characterization and Gas Sensing Measurements

The morphologies and compositions of as-prepared products were investigated by a scanning electron microscope (SEM, Zeiss Gemini 300) equipped with energy dispersive X-ray (EDX) spectroscope and a high-resolution transmission electron microscope (HRTEM, FEI Tecnai G2 F30). The chemical states of the surface species were determined by using X-ray photoelectron spectroscopy (XPS, ESCALB 250Xi). The gas sensing properties of sensors were performed on a commercial CGS-4TPs system (Beijing Elite Tech Co., Ltd., China). Gaseous acetone diluted with dry air was injected by a syringe. The operating temperature ranges from 280 to 380°C with a relative humidity around 20%.

## Figures and Tables

**Table 1:** Comparison of the acetone sensing properties of MOX-based sensors.

Materials	Temperature (°C)	RH (%)	Detection range (ppm)	Response (*R*_a_/*R*_g_)	*t*_res_/*t*_rec_ (s/s)	Ref.
*α*-Fe_2_O_3_/SnO_2_ HNAs	340	20	0.4–20	5.37@1 ppm	14/70	This work
Pt-SnO_2_ fibers	300	80	0.12–3	3.47@3 ppm	15/6	35
ZnO@ZIF-CoZn nanowire arrays	260	0–90	10–2000	2.3@10 ppm	43/61	36
PdO-Co_3_O_4_ hollow nanocages	350	90	0.4–5	1.52^∗^@1 ppm	—	37
NiO/ZnO hollow spheres	275	30	0.8–100	1.6@0.8 ppm	1/20	38
ZnO/CuO inverse opals	310	93.5	0.2–50	1.8@1 ppm	7/13	11

^∗^ *R*_g_/*R*_a_.

## Supplementary Materials

Supplementary Materials Figure S1: schematic drawing of the gas sensing measurement systems. Figure S2: SEM images of pure *α*-Fe_2_O_3_ NRs. Figure S3: transient response curve of *α*-Fe_2_O_3_ NRs toward different acetone concentrations at 340°C and the corresponding resistance curve. Figure S4: response/recovery time *vs.* acetone concentration (0.4-20 ppm) with SnO_2_ NSAs and *α*-Fe_2_O_3_/SnO_2_ HNAs at 340°C. Table S1: brief summary of results reported on exhaled breath analysis. Table S2: gas sensing properties of Fe_2_O_3_–SnO_2_ systems toward various gases.
