# Supplementary material for: Construction of 1D/2D α-Fe2O3/SnO2 Hybrid Nanoarrays for Sub-ppm Acetone Detection
Source: Research (Wash D C). 2020 Feb 13;2020:2196063. doi: 10.34133/2020/2196063 (PMC7040428; doi:10.34133/2020/2196063)
Supplement: Supplementary Materials — Figure S1: schematic drawing of the gas sensing measurement systems. Figure S2: SEM images of pure α-Fe2O3 NRs. Figure S3: transient response curve of α-Fe2O3 NRs toward different acetone concentrations at 340°C and the corresponding resistance curve. Figure S4: response/recovery time vs. acetone concentration (0.4-20 ppm) with SnO2 NSAs and α-Fe2O3/SnO2 HNAs at 340°C. Table S1: brief summary of results reported on exhaled breath analysis. Table S2: gas sensing properties of Fe2O3–SnO2 systems toward various gases. [file 2196063.f1.doc]

**Supporting Information**

**Construction of 1D/2D α-Fe_2_O_3_/SnO_2_ Hybrid Nanoarrays for Sub-ppm Acetone Detection**

Huimin Gong^1^, Changhui Zhao^1^,*, Gaoqiang Niu^1^, Wei Zhang^2^, and Fei Wang^1^,*

*^1^School of Microelectronics, Southern University of Science and Technology, Shenzhen, 518055, China*

*^2^Department of Electrical and Electronic Engineering, Southern University of Science and Technology, Shenzhen, 518055, China*

*Correspondence should be addressed to Fei Wang; wangf@sustech.edu.cn and Changhui Zhao; zhaoch@sustech.edu.cn

**TABLE S1:** Brief summary of results reported on exhaled breath analysis ^a^.

| Target disease | Target gas | Sensitive materials or technology | Concentration (ppb) | | Detection range (ppb) | Ref. |
| --- | --- | --- | --- | --- | --- | --- |
|  |  |  | Patient | Healthy |  |  |
| Lung cancer | Formaldehyde | Pd-SnO_2_ NPs | ~83 | ~48 | 30–1000 | 4 |
| Lung cancer | Toluene | Pd-WO_3_ NFs | 80–100 | 20–30 | 120–5000 | 5 |
| Hemodialysis | Ammonia | TFB | 2204 ± 1471 | 208 ± 301 | 30–2000 | 6 |
| Halitosis | H_2_S | Au-WO_3_ NPs | 80–2000 | 50-80 | 200–5000 | 7 |
| Heart disease | Isoprene | Pt-SnO_2_ | – | 22–234 | 5–500 | 8 |
| Smoker | Benzene | GC-MS | 14.05 | 1.15 | – | 9 |
| Asthma | Pentane | GC-MS | 190 ± 66 | 13–90 | – | 10 |

^a^ Abbreviations: NPs = nanoparticles, NFs = nanofibers, TFB = poly[(9,9-dioctylfluorenyl-2,7-diyl)-*co*-(4,4’-(*N*-(4-s-butylphenyl)diphenylamine)], GC-MS = gas chromatography-mass spectrometry.

**TABLE S2:** Gas-sensing properties of Fe_2_O_3_–SnO_2_ systems toward various gases.

| Sensitive materials **(**morphology**)** | Target gas | Concentration (ppm) | Temperature (°C) | Response (*R*_a_/*R*_g_) | Ref. |
| --- | --- | --- | --- | --- | --- |
| α-Fe_2_O_3_/SnO_2_ composites (α-Fe_2_O_3_ nanorods grow on SnO_2_ nanosheets) | acetone | 100 | 250 | 17 | [23](#_ENREF_1) |
| α-Fe_2_O_3_/SnO_2_ core-shell nanotubes (SnO_2_ nanoparticles coat on α-Fe_2_O_3_ nanotubes) | acetone | 100 | 300 | 33.4 | 24 |
| α-Fe_2_O_3_/SnO_2_ double-shell composites (α-Fe_2_O_3_ nanosheets on SnO_2_ hollow nanospheres) | ethanol | 100 | 225 | 18.4 | 25 |
| α-Fe_2_O_3_/SnO_2_ nanofibers (α-Fe_2_O_3_ nanoparticles decorate on SnO_2_ nanofibers) | ethanol | 100 | 300 | 22.46 | 26 |
| α-Fe_2_O_3_/SnO_2_ core-shell nanorods (SnO_2_ nanoparticles on α-Fe_2_O_3_ nanorods) | ethanol | 10 | 220 | 19.6 | 27 |
| SnO_2_/α-Fe_2_O_3_ nanotubes (SnO_2_ additives in α-Fe_2_O_3_ nanotubes) | ethanol | 100 | 200 | 27.45 | 28 |
| α-Fe_2_O_3_/SnO_2_ nanowires (α-Fe_2_O_3_ nanoparticles coat on SnO_2_ nanowires) | ethanol | 200 | 300 | 57.56 | 29 |
| hollow SnO_2_/α-Fe_2_O_3_ spheres (α-Fe_2_O_3_ nanorods grow on hollow SnO_2_ spheres) | ethanol | 100 | 250 | 16 | 30 |
| SnO_2_-Fe_2_O_3_ interconnected nanotubes (SnO_2_ nanotubes and α-Fe_2_O_3_ nanotubes) | toluene | 50 | 260 | 25.3 | 31 |
| α-Fe_2_O_3_/SnO_2_ thick films (α-Fe_2_O_3_ nanoparticles modify SnO_2_ hollow microspheres) | LPG | 1000 | 350 | 1991 | 32 |
| α-Fe_2_O_3_/SnO_2_ composites (α-Fe_2_O_3_ nanorods and SnO_2_ nanorods) | LPG | 10000 | 370 | ~32 | 33 |

**
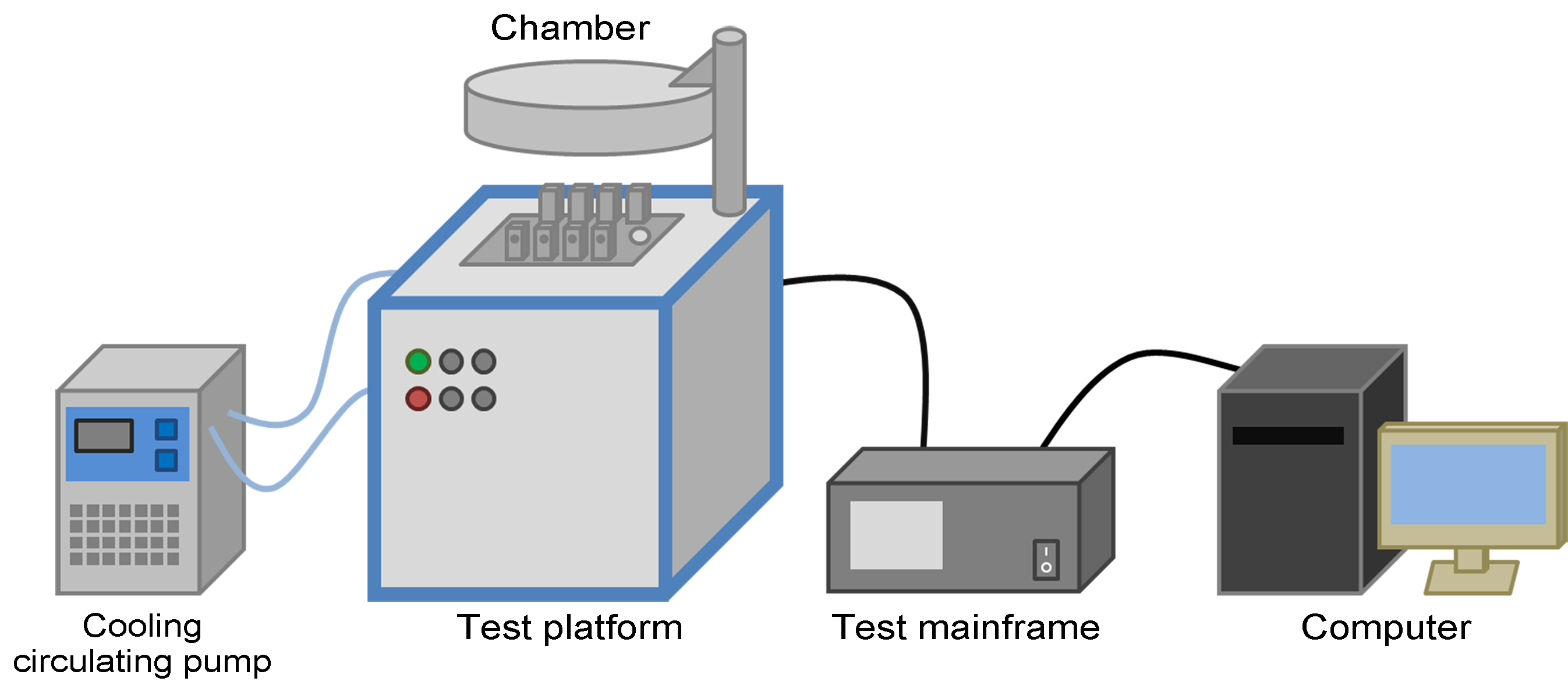
**

**FIGURE S1:** Schematic drawing of the gas-sensing measurement systems.


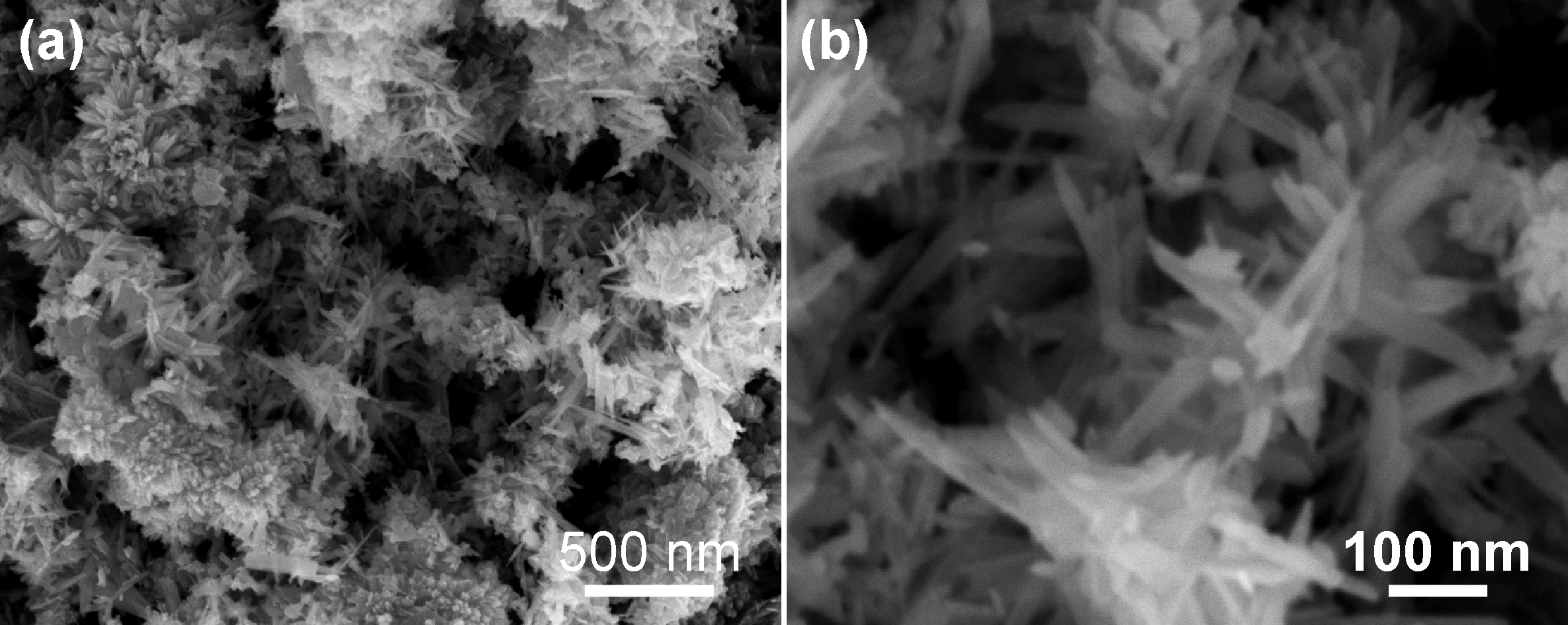


**FIGURE S2:** (a, b) SEM images of pure α-Fe_2_O_3_ NRs.


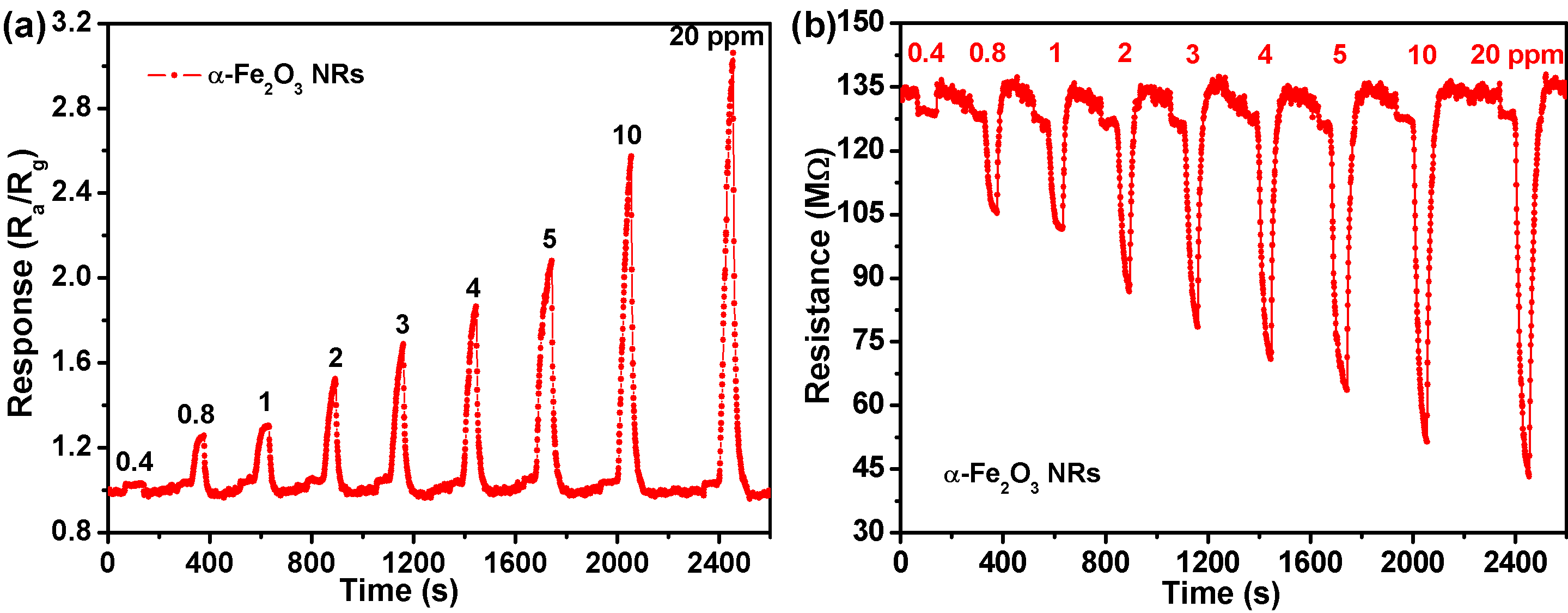


**FIGURE S3:** (a) Transient response curve of α-Fe_2_O_3_ NRs toward different acetone concentrations at 340 °C, and (b) the corresponding resistance curve.


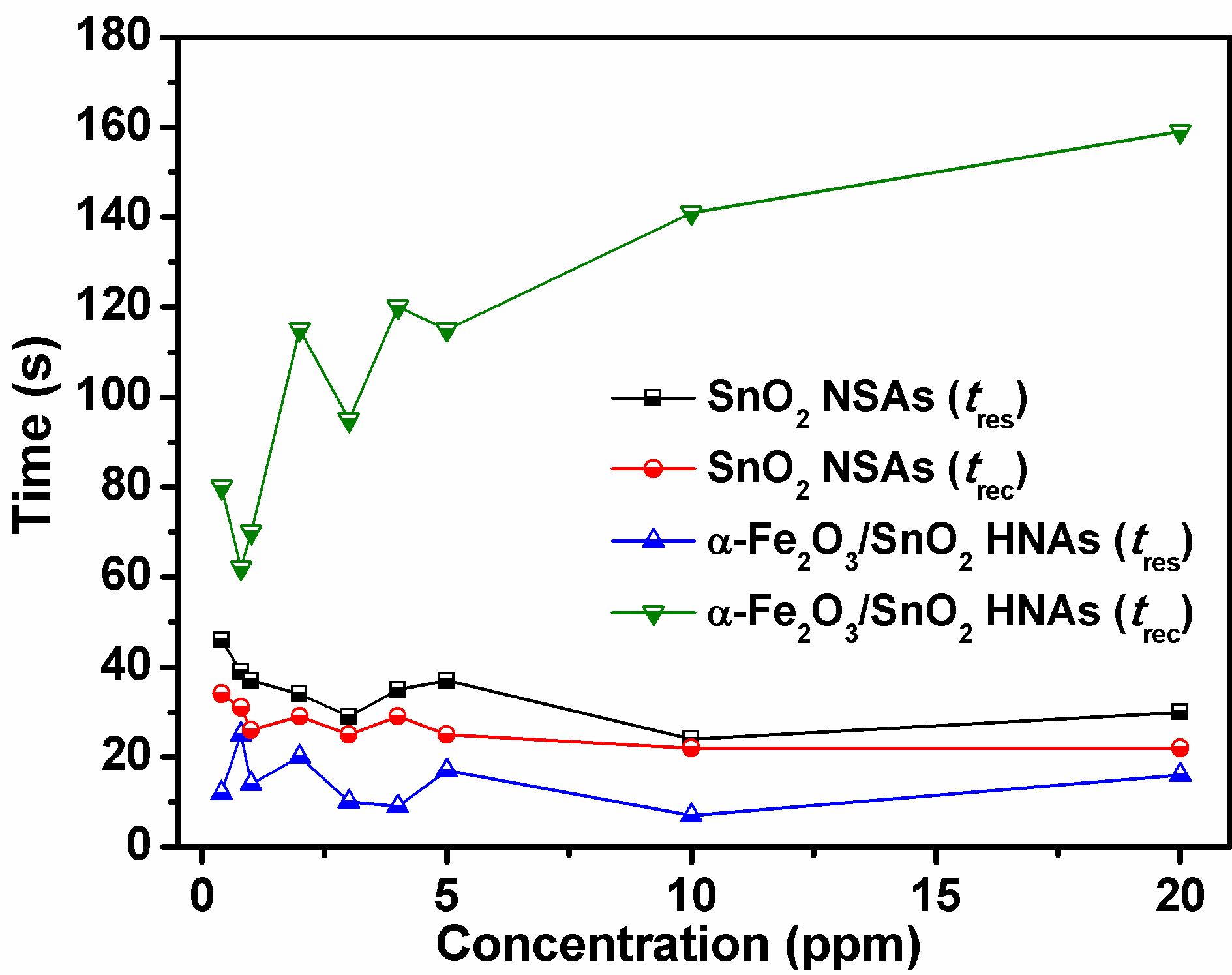


**FIGURE S4:** Response/recovery time *vs.* acetone concentration (0.4-20 ppm) with SnO_2_ NSAs and α-Fe_2_O_3_/SnO_2_ HNAs at 340 °C.
